# Including a discussion forum in a web-based intervention on fertility and sexuality following cancer – Usage and content

**DOI:** 10.1016/j.invent.2022.100559

**Published:** 2022-07-08

**Authors:** Maria Gottvall, Kristina Fagerkvist, Claudia Lampic, Lena Wettergren

**Affiliations:** a Department of health Sciences, The Swedish Red Cross University College, Box 1059, SE-141 21 Huddinge, Sweden; b Department of Women's and Children's Health, Uppsala University, SE-751 85 Uppsala, Sweden; c Department of Women's and Children's Health, Karolinska Institutet, Tomtebodavägen 18A, SE-171 77 Stockholm, Sweden; d Centre for Clinical Research Sörmland, Uppsala University, SE-631 88 Eskilstuna, Sweden; e Department of Psychology, Umeå University, SE-901 87 Umeå, Sweden; f Department of Public Health and Caring Sciences, Uppsala University, Box 564, SE-751 22 Uppsala, Sweden

**Keywords:** Childhood cancer survivors, Communication, Fertility, Online discussion forum, Sexuality, Social support

## Abstract

**Aim:**

The aim of the study was to investigate how young adult survivors of childhood cancer used an online discussion forum as part of a web-based psycho-educational intervention. Specifically, we aimed to characterize users of the discussion forum, investigate how they used the discussion forum (type of usage) and content of the posted messages.

**Methods:**

This study is a part of a randomized controlled trial, Fex-Can Childhood RCT. Participants with self-reported sexual dysfunction or fertility-related distress were drawn from a population-based national cohort. Sociodemographic and clinical characteristics of the intervention group (n = 322) and data on usage of the discussion forum were analysed with descriptive statistics and compared between subgroups. Messages posted in the online discussion forum were analysed with qualitative thematic analysis.

**Results:**

Approximately half (48 %) of participants in the intervention group accessed the discussion forum and most of them (76 %) without writing own posts. Users of the discussion forum did not statistically differ in sociodemographic or clinical characteristics from the rest of the intervention group. The 97 written posts, written by 38 individuals, were mainly descriptions of own experiences and thoughts and concerned three themes: *A changed body*, *Concerns around family building* and *Longing for support*. Peer-support and interaction between participants were seen in some forum threads and the ‘like’-function was frequently used, demonstrating engagement and activity. Participants expressed that they felt affinity with and appreciated sharing own experiences and to recognize themselves in others' stories.

**Conclusions:**

A discussion forum as part of a web-based intervention appears to be a valuable component by giving participants an opportunity to share intimate experiences and concerns related to surviving cancer.

**Trial registration:**

ISRCTN Registry, trial number: 33081791 (registered on November 27, 2019).

## Highlights

• Half of the intervention group accessed the discussion forum to read or write posts.
• A small proportion of those who accessed the discussion forum wrote own posts.
• Participants shared experiences related to sex life, intimacy and family building.
• Participants felt affinity with their peers by reading posts in the discussion forum.

## 1 Introduction

Approximately half a million people in Europe are living with an experience of childhood cancer (Childhood Cancer International - Europe, 2021). Even though most children and teenagers treated for cancer will survive, many of them risk late effects related to their malignancy or its treatment (Langer et al., 2017). Long-term consequences include negative impact on sex life and fertile ability as well as concerns related to these areas (Newton et al., 2019; Logan et al., 2019). According to a recent nationwide study of young adult survivors of childhood cancer (aged 19–40), more than half of women and approximately one third of men, experience sexual dysfunction (Hovén et al., 2021). Common reported problems were low interest in having sex, difficulties reaching orgasm, low satisfaction with sex life and erectile dysfunction. Cancer treatment during childhood may also cause hormonal disorders in both females and males that may have a negative impact on future fertility (Newton et al., 2019; Green et al., 2010; Green et al., 2009; Rose et al., 2016). In addition, young adult childhood cancer survivors report that scars and other physical late effects of the treatment, and fertility concerns may have a negative impact on their romantic relationships (Nahata et al., 2020).

Several web-based interventions have been developed to support people diagnosed with cancer, but few address sexual and fertility-related problems. Web-based interventions typically include information as well as behavioural change content and can have a more or less automatized format (Barak et al., 2009; Pingree et al., 2010). Evaluations of interventions targeting sexual and fertility issues with randomized controlled or pre- and post-test designs have shown some positive effects. For example, improved overall sexual function (Hummel et al., 2017; Schover et al., 2020; Wootten et al., 2017), self-esteem (Hummel et al., 2017), body image (Wootten et al., 2017), and cancer-related fertility knowledge (Micaux et al., 2022) as well as a decrease in fertility concerns (Irene Su et al., 2019). Three of these interventions, directed to persons diagnosed with cancer as adults included a discussion forum with the purpose to increase engagement and activity (Classen et al., 2013; Wootten et al., 2017; Micaux et al., 2022). These studies included participants with lymphoma, gynecological-, prostate-, breast-, testicular cancer, and Central nervous system (CNS) tumours. The only study that tested the possible effect of including a discussion forum found that this improved sexual function compared to receiving the intervention without a forum (Wootten et al., 2017). Having access to an online forum as part of a web-based intervention targeting sexual and fertility issues enables discussion of sensitive topics while remaining anonymous, which is valued by participants (Classen et al., 2013; Wiljer et al., 2011). Furthermore, peer support is suggested to increase participants' sense of normalcy and reduce feelings of loneliness (Micaux Obol et al., 2020).

In conclusion, a discussion forum may be a valuable component in web-based interventions targeting sexuality and fertility, but its contribution is not fully understood. Based on the lack of interventions focused on young adults with a cancer experience the “Fex-Can Childhood” project was initiated to address potential problems related to sexual and reproductive health in childhood cancer survivors (Ljungman et al., 2020). It consists of a nationwide observational study with an embedded randomized controlled trial (RCT) evaluating a web-based psycho-educational intervention, including a discussion forum. In the present study we aim to investigate how young adult childhood cancer survivors use a discussion forum within a web-based psycho-educational intervention. More specifically, we aimed to characterize users of the discussion forum, investigate how they used the discussion forum (type of usage) and content of the posted messages.

## 2 Methods

The present study is part of the Fex-Can Childhood project (Ljungman et al., 2020). The observational study aimed to investigate sexual function and fertility-related distress in a national cohort of childhood cancer survivors, aged 19–40 at the time of the study. In oncology, young adults are commonly defined as 19–39 years of age (“What Should the Age Range Be for AYA Oncology?,” 2011). All participants who rated sexual dysfunction or fertility-related distress, according to pre-determined definitions, were invited to participate in an RCT to evaluate the effect of the web-based psycho-educational intervention including an online moderated discussion forum. The primary outcomes of the RCT were ‘Satisfaction with sex life’ and ‘Fertility rated distress’. The study is registered in the ISRCTN Registry (33081791).

### 2.1 Participants

**Fig. 1:**
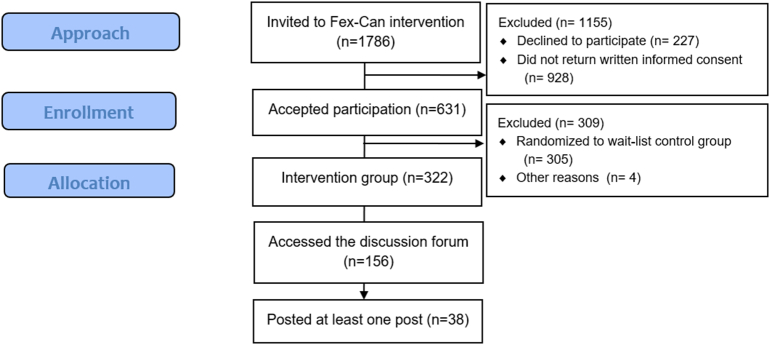
Flow diagram of study participants.

Among those invited to the intervention study, 631 women and men accepted participation and were subsequently randomized to the intervention group (IG) or a wait-list control group (Fig. 1). Participants in the IG had access to an online moderated discussion forum. The present study is based on the IG (n = 322) including those who actively used the discussion forum, hereafter called ‘forum users’ (n = 156), as well as those who wrote at least one post in the discussion forum, hereafter called ‘posters’ (n = 38).

### 2.2 Psycho-educational intervention

The web-based psycho-educational intervention was developed in cooperation with young adults with a cancer experience and mothers to teenagers treated for cancer (Winterling et al., 2016). It was delivered as two programs, Fex-Can Sex targeting sexual problems, and Fex-Can Fertility targeting concerns about fertility (Ljungman et al., 2020). Each program consisted of six modules (see Table 1), delivered every other week during a period of 12 weeks. The modules dealt with specific topics concerning sexuality or fertility and comprised of information such as how different cancer treatments could affect sex life and fertility. The programs also included videos and quotes from young adult survivors of childhood cancer sharing their experiences regarding these areas, and exercises to explore physical contact alone or together with someone as well as mindfulness-exercises. Additionally, the two programs shared an online discussion forum, moderated by two research group members with experience from cancer care. The moderators monitored the discussion forum on a daily basis and their main role was to identify if any of the participants' posts indicated emotional distress or disease-related problems meriting concern. In the discussion forum, participants could interact with each other asynchronously by writing own posts, ‘liking’ (in the shape of a heart) others' posts, and replying to each other's posts (Ljungman et al., 2020). Participants as well as moderators could start discussion threads in the forum. After the 12 weeks, participants had access to the intervention including the discussion forum for another two weeks.

**Table 1:** Overview of the modules included in the two Fex-Can intervention programs.

Fex-Can Sex		Fex-Can Fertility
Introduction to sexuality	Shared discussion forum	Introduction to fertility following cancer
Lack of desire		Handling distress
Discomfort and pain (females) Erection (males)		Trying to achieve a pregnancy
Orgasm (females) Orgasm and ejaculation (males)		Child and personal health
Relations and sex		Alternative ways to build a family
My body		Relationships

### 2.3 Data collection and analysis

#### 2.3.1 Characteristics of forum users and posters

Sociodemographic characteristics of the participants were based on survey data from the observational study (birth country, education, occupation, partner, children, sexual orientation). Clinical data were retrieved from the National Quality Registry for Childhood Cancer Register (diagnosis, treatment, age at diagnosis and study, time since diagnosis). Chi-square tests, Fischer's Exact tests, and independent *t*-tests were performed to compare subgroups (‘posters’ vs. ‘non-posters’; ‘forum users’ vs. ‘non-forum users’).

#### 2.3.2 Type of usage

Data on usage of the discussion forum were extracted from the website system and included: accessing the forum (yes/no), time spent in the forum (minutes), providing ‘likes’ to other posts (frequency), number of written posts. Forum users were defined as participants spending at least 0.5 min in the discussion forum, estimated as the minimum time needed to read and/or ‘like’ a post. Mann-Whitney *U* test was performed to compare ‘posters’ vs. ‘non-posters’.

#### 2.3.3 Content of posts

All written posts in the discussion forum were extracted from the website system. The content of the posts were analysed with inductive qualitative thematic analysis (Braun and Clarke, 2006). Inductive thematic analysis is a useful and flexible method for analysing various types of qualitative data, holding the themes closely linked to the data (data-driven analysis) and exploring the data without being bound to a specific pre-existing theoretical framework. Coding of the data set was performed manually by writing notes on the text. The codes were then sorted into potential themes. The pattern of the codes as well as the names/validity of the themes and sub-themes were discussed among co-authors until all agreed that the themes captured the contours of the coded data. During the whole analysis process, the data set was reread several times. In addition, all the posts were classified as either a description of own experiences and thoughts, a question phrased to other participants, or a direct reference or response to another person's post.

### 2.4 Ethical considerations

Ethical approval for the Fex-Can Childhood study was obtained by the Regional Ethical Review Board in Stockholm, Sweden (Dnr: 2015/1609-31; 2018/2688-32; 2019/01066; 2019/04603). Eligible participants received written information about the study and its purpose, that participation was voluntary and could be withdrawn at any time. Written informed consent was obtained from all participants. To receive access to the program participants logged in with their email address and a self-selected password. To post something in the discussion forum they had to choose an alias, in this way the participants were anonymous to other intervention participants. At the stage of analysing the data, researchers connected participants' alias with their code. Data were processed according to The EU General Data Protection Regulation (GDPR).

## 3 Results

### 3.1 Characteristics of forum users and posters

Approximately half of the intervention group had used the discussion forum (‘forum users’) and 38 of them had written posts (‘posters’). The average age of the ‘forum users’ was 29 years and a majority had received their cancer diagnosis before turning thirteen (see Table 2). Approximately 70 % of the ‘forum users’ were women, had a partner, and one third had children. Comparisons of subgroups (‘forum users’ vs. ‘non-users’; ‘posters’ vs. ‘non-posters’) showed no statistically significant differences with regard to sociodemographic and clinical characteristics (see Table 2, statistical results not shown).

**Table 2:** Characteristics of participants in the intervention group, forum users, and posters in the discussion forum.

	Intervention group (n = 322)	Forum users (n = 156)	Posters (n = 38)
	No. (%)	No. (%)	No. (%)
*Sociodemographics*			
Age at study, years			
Mean (SD)	29.0 (5.8)	29.3 (5.8)	30.1 (5.5)
Gender			
Women	208 (64.6)	108 (69.2)	30 (78.9)
Men	114 (35.4)	48 (30.8)	8 (21.1)
Country of birth			
Sweden	312 (97.2)	153 (98.1)	38 (100.0)
Other	9 (2.8)	3 (1.9)	0 (0.0)
Education			
Elementary	6 (1.9)	2 (1.3)	1 (2.6)
Upper secondary	110 (34.3)	49 (31.4)	9 (23.7)
University	190 (59.2)	94 (60.3)	26 (68.4)
Other	15 (4.7)	11 (7.1)	2 (5.3)
Occupation			
Working/studying	282 (87.6)	137 (87.8)	34 (89.5)
Other^a^	40 (12.4)	19 (12.2)	4 (10.5)
Partnered	220 (68.5)	108 (69.2)	29 (76.3)
Have children	95 (29.6)	46 (29.5)	10 (26.3)
Sexual orientation^b^			
Heterosexual	287 (90.0)	142 (91.0)	36 (94.7)
Non-heterosexual^c^	32 (10.0)	14 (9.0)	2 (5.3)

*Clinical characteristics*			
Age at diagnosis, years			
Mean (SD)	7.3 (5.5)	7.4 (5.6)	7.0 (5.1)
0–5	149 (51.6)	72 (46.2)	18 (47.4)
6–12	72 (22.4)	45 (28.8)	12 (31.6)
13–17	78 (24.2)	39 (25.0)	8 (21.1)
Time from diagnosis, years			
Mean (SD)	21.2 (7.4)	21.3 (7.5)	22.6 (6.2)
Range	1–37	1–37	9–36
Type of cancer^d^			
Haematological	166 (51.6)	79 (50.6)	21 (55.3)
CNS tumours	72 (22.4)	30 (24.4)	7 (18.4)
Solid tumours	24 (26.1)	39 (25.0)	10 (26.3)
Treatment modality			
Chemotherapy	241 (86.7)	117 (75.0)	29 (76.3)
Surgery	97 (30.1)	54 (34.6)	11 (28.9)
Radiotherapy	70 (69.3)	37 (23.7)	7 (18.4)
Stem cell transplantation	31 (9.6)	13 (8.3)	3 (7.9)

CNS, central nervous system.

a Includes individuals who reported being unemployed, sick-leave, parental leave.

b 3 women in the intervention group preferred not to answer.

c Includes individuals who reported not knowing, being pansexual, queer, or asexual.

d According to the International Classification of Childhood Cancer, third edition (ICCC-3).

### 3.2 Type of usage

Almost half (n = 156, 48 %) of the participants accessed the discussion forum at least once during the intervention. A majority of the ‘forum users’ (n = 118, 76 %) spent time in the discussion forum without writing any posts. In total, there were 97 posts in the discussion forum distributed among the 38 individuals (30 women and 8 men) who posted. The ‘posters’ spent significantly more time (md = 42.6 min, IQR = 55.4) in the discussion forum than the ‘non-posters’ (md = 2.0 min, IQR = 5.1, p < 0.001). A total of 343 ‘likes’ were provided to posts in the forum. The ‘posters’ to a larger extent gave ‘likes’ than the ‘non-posters’ (70.3 % vs. 29.3 %, p < 0.001), with a median of 1.5 ‘likes’ (IQR = 6) compared to a median of 0 ‘likes’ (IQR = 0, p ≤ 0.001).

### 3.3 Content of the posts in the discussion forum

The vast majority of the 97 messages posted in the discussion forum concerned participants' own experiences and thoughts (n = 80, 82 %). Remaining posts included questions or inquiries about other participants' experiences (n = 8, 8 %), or direct references or answers to someone else's post (n = 9, 9 %).

**Fig. 2:**
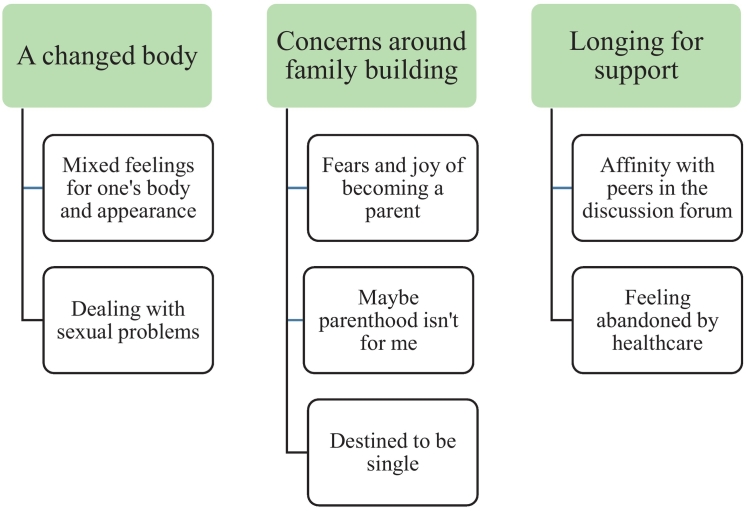
Themes and sub-themes identified in the analysis.

Analysis of the posts resulted in three main themes: *A changed body*, *Concerns around family building* and *Longing for support*. Each theme consists of two to three sub-themes (Fig. 2), which are presented below and illustrated by quotes.

#### 3.3.1 A changed body

##### 3.3.1.1 Mixed feelings for one's body and appearance

Physical appearance was described as an important issue and participants expressed that the cancer and its treatment had had a large impact on how they viewed themselves and their bodies. Several participants expressed an overall difficult relation to one's body and increased attention to any physical symptoms of disease as they were worried about relapse. Other posts concerned treatment-related changes of physical appearance, such as loss of hair and treatment-related swelling, which sometimes had led to being bullied. Even though it was many years since they were diagnosed and underwent treatment, these experiences could still affect their self-esteem and how they viewed themselves. Participants also described a complicated relation to food with fear of gaining weight. Furthermore, they expressed that spending a lot of time in treatment and at the hospital had given them a sense of ‘not owning their own body’, which is illustrated by the quote below.

“At times, it washes over me that I did not own my own body. I remember being physically restrained [at the hospital] and information about increased risk of becoming overweight created a fear of food… So, the love for one's own body is difficult”

(Woman, 33 years, <13 years old at diagnosis)

Different views on surgery scars were prominent in the discussion forum. Many participants described that they felt ashamed and less attractive because of their scars, and some shared strategies to avoid showing them, for example covering scars with tattoos or avoiding being undressed around other people. Others posted that they felt proud of their scars and of a body that had survived cancer, affecting self-esteem and sexuality positively.

“My scar was the size of a large coin. I was very ashamed and was always told that it was disgusting, ugly, looked like a hickey and so on. I used to buy clothes that hid it to avoid comments. When I was 17, I decided to cover the scar with a tattoo and it was the best decision of my life! So proud of my tattoo and my self-esteem took a giant step up!”

(Woman, 25 years, <13 years old at diagnosis)

##### 3.3.1.2 Dealing with sexual problems

Some participants had not reflected over the possibility that cancer could impact on adult sex life. However, several participants posted messages describing sex problems and dissatisfaction with their sex life. Feelings of inadequacy in sexual relations were described and related to fatigue, as well as vaginal dryness, and low libido. A strategy to find sexual pleasure could be to test new partners, as described in the quote below.

“The first years I had big problems with sex as my body simply was too tired, had no problems having intercourse or so but had big problems feeling any pleasure in it all. This contributed to me having several different sex partners in parallel as I wanted to feel something, I changed partners often. It was not until I got someone to talk to, the right help with physiotherapy and diet that I could actually enjoy sex”

(Man, 38 years, ≥13 years old at diagnosis)

#### 3.3.2 Concerns around family building

##### 3.3.2.1 Fears and joy of becoming a parent

Several posts described a strong desire to have children and worries about one's fertile ability. Some participants shared intimate stories of their struggle to become pregnant, miscarriages and complicated pregnancies.

“For me, being able to have children was never a certainty… It took some time. Today I have three children… I went to extra checks during the pregnancy since they were afraid that I might have a heart failure during childbirth and the pregnancies were so close. I am feeling really lucky today.”

(Woman, 39 years, <13 years old at diagnosis)

Participants who had children expressed joy and happiness related to their parenthood, but also perceived themselves to be more worried than other parents. One of their greatest fears seemed to be that their own child/children would get severely ill.

##### 3.3.2.2 Maybe parenthood isn't for me

Another perspective on parenthood expressed in the discussion forum concerned a reluctance to have children, that parenthood maybe was not for them. Participants' doubts about becoming a parent were related to uncertainty if their body could manage a pregnancy, being too tired to take care of a child, and being afraid of getting cancer again. One young man (24 years, ≥13 years old at diagnosis) described that he had frozen sperm to use in the future if needed, but expressed hesitation to use them because of his fear of relapse and early death.

Other posts described having no desire to become a parent. As illustrated in the conversation below, this was perceived as an uncommon stance, reflected upon as a deliberate choice, a personality trait or a defense mechanism in the face of physical obstacles to raise a child.

“I don't want to sacrifice my life to another human being, which having children seems to mean. I want to live the life that I got back, I want to travel, realize dreams and life goals. I don't want to say no, have to think of someone else or live my life according to someone else's needs. But everyone I've dated, everyone at work and all my friends, no one thinks like me. Therefore, I wonder if I am the only one having these feelings?”

(Woman, 25 years, <13 years old at diagnosis)

“Hi, No you're not alone. I have never had a strong desire to start a family. I don't know if it's a defense mechanism that sets in motion because I probably would not be physically able to take care of children, or if it's because I am the person I am.”

(Woman, 39 years, ≥13 years old at diagnosis)

##### 3.3.2.3 Destined to be single

Several participants wrote that they were too tired to be in a relationship. Furthermore, another reason expressed for remaining single, was the fear of seeing a potential partner suffer in the case of the cancer reoccurring. Mental ill health, such as depression, was also mentioned as an explanation for being single.

“I have always lived alone and not been well enough to feel that I would be able to live with someone or take care of children.”

(Woman, 39 years, ≥13 years old at diagnosis)

#### 3.3.3 Longing for support

##### 3.3.3.1 Affinity with peers in the discussion forum

Participants expressed that it felt good to share their own experiences and feelings, and also to recognize themselves in stories told by other participants. During the time of cancer treatment, the participants often felt lonely around other people who did not share similar experiences and they therefore kept their cancer experience secret. People with comparable experiences, as in the discussion forum, were perceived as being able to give more support.

“…it is strangely comforting to know that there are others who have similar experiences. Before, I was completely alone with everything but now I understand that there's nothing wrong about how I think/feel.”

(Woman, 35 years, <13 years old at diagnosis)

##### 3.3.3.2 Feeling abandoned by healthcare

Several participants expressed that they felt abandoned by health care after the cancer treatment was over. Participants also expressed unmet needs regarding psychological support and information about fertility- and sexuality-related issues, both during and after treatment. This is illustrated by two quotes from a conversation with several posts.

“I have never been offered psychological help after the treatment was over. Only during it and at that time I was not receptive. No follow-up has ever been conducted. Are there others who experience the same thing?”

(Woman, 34 years, ≥13 years old at diagnosis)

“I absolutely believe that everything around your treatment and ‘living’ with cancer has affected one's life and who you are today. What you say about never having been offered psychological help is what I mean by them ‘forgetting’ us as soon as the treatment is finished, and you should be ‘well’.”

(Man, 38 years, ≥13 years old at diagnosis)

## 4 Discussion

The present study investigated how young adult survivors of childhood cancer used a discussion forum within a web-based intervention targeting reproductive and sexual issues. Our results show that about half of the participants in the intervention accessed the discussion forum and participants expressed that they felt affinity with and appreciated the support from peers in the forum. Most of the posted messages in the forum were descriptions of own experiences and the content of the posts are presented in the three themes *A changed body*, *Concerns around family building*, and *Longing for support*.

What characterizes those who are active users in a discussion forum within a web-based intervention? Those who used the discussion forum and those who posted messages did not differ statistically significantly regarding sociodemographic and clinical characteristics compared to the rest of the intervention group, but parts of the analyses had low power. Still, it appears that a discussion forum as part of a web-based intervention for young childhood cancer survivors attracts similar participants as those choosing to take part in the intervention. The study participants were to a great extent women and people with university education, which corresponds to previous research about participation in surveys among childhood cancer survivors (Kilsdonk et al., 2017). The low proportion of men actively participating in psycho-educational interventions targeting female and male cancer patients has been previously reported (Micaux et al., 2022).

Participants expressed satisfaction with having access to the discussion forum as it gave them a feeling of affinity. Hearing about other childhood cancer survivors' lives, worries, thoughts and problems made them feel less alone and increased their sense of normalcy. This highlights the importance of relatedness, to experience connection with others, which is a basic psychological need according to the self-determination theory (Ryan and Deci, 2000). These findings are also in line with other studies showing that web-based interventions provide a supportive environment for people with cancer and can reduce feelings of loneliness (Micaux Obol et al., 2020; Wiljer et al., 2011). Emotional online support from peers sharing similar experiences is valued by childhood cancer survivors (Nilsson et al., 2022). Interestingly, one study found a significant effect on overall sexual satisfaction in men with localized prostate cancer when including a discussion forum in an online psychological intervention (Wootten et al., 2017). In the present study, a small proportion of those who had access to the web-based intervention (38 out of 322 individuals in the IG) contributed with own posts in the discussion forum. While they most often wrote posts in the forum to share their own experiences, peer-support and interaction between the participants could be seen in some forum threads and the ‘like’-function was frequently used. We also saw that there were participants who spent time reading posts in the discussion forum without writing own posts, in line with previous studies (Classen et al., 2013; Micaux Obol et al., 2020; Wootten et al., 2017). Whether the number of posted messages and the number of participants accessing the discussion forum is high or low is difficult to say since few intervention studies with a discussion forum have presented such data (Classen et al., 2013; Wootten et al., 2017). Possible ways to increase activity in a discussion forum suggested by Classen et al. (2013) include encouraging participants to write at least one post every week, presenting pre-defined topics for discussion, and contacting each participant at study start.

Sensitive and private descriptions related to a changed body and sex life were posted in the discussion forum of the present study. Childhood cancer survivors' concerns related to changes of the physical body have been reported in previous studies and include for example difficulties with having visible scars (Jervaeus et al., 2016; Nahata et al., 2020; Olsson et al., 2018). In the present study, feeling ashamed of visible scars was discussed and creative strategies to hide them were mentioned, such as covering the scar with a tattoo. In addition, feelings of not owning their own body had negatively affected participants' self-esteem. The option to remain anonymous when writing and responding to posts is believed to have facilitated participants' communication of thoughts and experiences regarding sensitive topics, as also previously suggested (Classen et al., 2013).

Posts in the discussion forum reflected a wide range of perspectives regarding parenthood following cancer. Our findings are in line with previous reports that many childhood cancer survivors want to have children (van Dijk et al., 2018) and that worries about possible infertility are common (Lehman et al., 2019; Logan et al., 2019; Nilsson et al., 2014). Descriptions of grief related to potential fertility loss and conditional joy when finally becoming pregnant (Vanstone et al., 2021), were also identified in the present study. In line with Lehman et al. (Lehman et al., 2019) our results show that there is a group of childhood cancer survivors who do not want to have children. Some expressed that this was because they were too tired to have children, and some were even too tired to be in a relationship. Thus, a preference for not having children could be related to limited physical capacity after cancer treatment, but it could also be a personal choice not related to the cancer experience, in line with previous findings (Zebrack et al., 2004).

### 4.1 Methodological considerations

This study has several strengths but also some limitations to be considered when interpreting the results. First, the large sample based on a national cohort of childhood cancer survivors (Hovén et al., 2021; Ljungman et al., 2020) is a strength. However, men are underrepresented in the study and the rather small proportion of ‘posters’ decreased the power in subgroup analyses. Second, the online delivery of the intervention facilitated inclusion of participants from all over the country. Third, having two moderators monitoring the discussion forum was beneficial to detect if any of the participants would indicate serious psychological problems. Additionally, data were available on both sociodemographic and clinical characteristics of all participants, which allowed us to investigate if these variables influenced the usage of the discussion forum. Finally, the results concerning the content of the posts in the discussion forum should be regarded within the context of the focus of the web-based intervention directed to childhood cancer survivors rating sexual dysfunction and/or fertility-related distress (Ljungman et al., 2020). Unfortunately, barriers to posting in the discussion forum were not studied and are recommended to be investigated in the future.

## 5 Conclusion

An opportunity to communicate with others sharing similar experiences through a discussion forum as part of a web-based intervention appears to be valuable for childhood cancer survivors. The forum creates a sense of belonging and confirmation that one's feelings and experiences are understandable and common. eHealth interventions combining educational information with a moderated discussion forum are recommended to young adult cancer survivors.

## Funding

This study was funded by the 10.13039/501100002794Swedish Cancer Society (CAN 2013/886); the 10.13039/501100006313Swedish Childhood Cancer Foundation (TJ2014-0050, TJ2019-0045, PR2014-0177, PR2016-0075 and PR2017-0037); the 10.13039/501100006636Swedish Research Council for Health, Working Life and Welfare (2014-4689), the 10.13039/501100004359Swedish Research Council (2017-01530), the 10.13039/501100004047Karolinska Institutet Faculty Funds (2-5586/2017) and the Centre for Clinical Research Sörmland (10.13039/501100007051Uppsala University). The funding sources were not involved in the design or conduction of the study.

## Declaration of competing interest

The authors declare that they have no known competing financial interests or personal relationships that could have appeared to influence the work reported in this paper.

## References

[bb0005] Barak A., Klein B., Proudfoot J.G. (2009). Defining internet-supported therapeutic interventions. Ann. Behav. Med..

[bb0010] Braun V., Clarke V. (2006). Using thematic analysis in psychology. Qual. Res. Psychol..

[bb0015] Childhood Cancer International Europe (2021). Childhood cancer international- Europe [CCIE] [Online]. https://ccieurope.eu/.

[bb0020] Classen C.C., Chivers M.L., Urowitz S., Barbera L., Wiljer D., O'Rinn S., Ferguson S.E. (2013). Psychosexual distress in women with gynecologic cancer: a feasibility study of an online support group. Psychooncology.

[bb0025] Green D.M., Kawashima T., Stovall M., Leisenring W., Sklar C.A., Mertens A.C., Donaldson S.S., Byrne J., Robison L.L. (2009). Fertility of female survivors of childhood cancer: a report from the childhood cancer survivor study. J. Clin. Oncol..

[bb0030] Green D.M., Kawashima T., Stovall M., Leisenring W., Sklar C.A., Mertens A.C., Donaldson S.S., Byrne J., Robison L.L. (2010). Fertility of male survivors of childhood cancer: a report from the childhood cancer survivor study. J. Clin. Oncol..

[bb0035] Hovén E., Fagerkvist K., Jahnukainen K., Ljungman L., Lähteenmäki P.M., Axelsson O., Lampic C., Wettergren L. (2021). Sexual dysfunction in young adult survivors of childhood cancer - A population-based study. Eur. J. Cancer.

[bb0040] Hummel S.B., van Lankveld J., Oldenburg H.S.A., Hahn D.E.E., Kieffer J.M., Gerritsma M.A., Kuenen M.A., Bijker N., Borgstein P.J., Heuff G., Lopes Cardozo A.M.F., Plaisier P.W., Rijna H., van der Meij S., van Dulken E.J., Vrouenraets B.C., Broomans E., Aaronson N.K. (2017). Efficacy of internet-based cognitive behavioral therapy in improving sexual functioning of breast cancer survivors: Results of a randomized controlled trial. J. Clin. Oncol..

[bb0045] Irene Su H., Stark S., Kwan B., Boles S., Chingos D., Ehren J., Gorman J.R., Krychman M., Romero S.A.D., Mao J.J., Pierce J.P., Natarajan L. (2019). Efficacy of a web-based women's health survivorship care plan for young breast cancer survivors: a randomized controlled trial. Breast Cancer Res Treat.

[bb0050] Jervaeus A., Nilsson J., Eriksson L.E., Lampic C., Widmark C., Wettergren L. (2016). Exploring childhood cancer survivors' views about sex and sexual experiences -findings from online focus group discussions. Eur. J. Oncol. Nurs..

[bb0055] Kilsdonk E., Wendel E., van Dulmen-den Broeder E., van Leeuwen F.E., van den Berg M.H., Jaspers M.W. (2017). Participation rates of childhood cancer survivors to self-administered questionnaires: a systematic review. Eur. J. Cancer Care (Engl.).

[bb0060] Langer T., Grabow D., Steinmann D., Wormann B., Calaminus G. (2017). Late effects and long-term follow-up after cancer in childhood. Oncol. Res. Treat..

[bb0065] Lehman V., Ferrante A.C., Winning A.M., Gerhardt C.A. (2019). The perceived impact of infertility on romantic relationships and singlehood among adult survivors of childhood cancer. Psycho-Oncology.

[bb0070] Ljungman L., Anandavadivelan P., Jahnukainen K., Lampic C., Wettergren L. (2020). Study protocol for the fex-can childhood project: An observational study and a randomized controlled trial focusing on sexual dysfunction and fertility-related distress in young adult survivors of childhood cancer. Medicine (Baltimore).

[bb0075] Logan S., Perz J., Ussher J.M., Peate M., Anazodo A. (2019). Systematic review of fertility-related psychological distress in cancer patients: Informing on an improved model of care. Psychooncology.

[bb0080] Micaux C., Wiklander M., Eriksson L.E., Wettergren L., Lampic C. (2022). Efficacy of fex-can fertility, a self-help web-based psychoeducational intervention for young adults with fertility distress following cancer – randomized controlled trial. JMIR Cancer.

[bb0085] Micaux Obol C., Lampic C., Wettergren L., Ljungman L., Eriksson L.E. (2020). Experiences of a web-based psycho-educational intervention targeting sexual dysfunction and fertility distress in young adults with cancer-A self-determination theory perspective. PLoS One.

[bb0090] Nahata L., Morgan T.L., Lipak K.G., Olshefski R.S., Gerhardt C.A., Lehmann V. (2020). Romantic relationships and physical intimacy among survivors of childhood cancer. J. Adolesc. Young Adult Oncol..

[bb0095] Newton H.L., Friend A.J., Feltbower R., Hayden C.J., Picton H.M., Glaser A.W. (2019). Survival from cancer in young people: An overview of late effects focusing on reproductive health. Acta Obstet. Gynecol. Scand..

[bb0100] Nilsson J., Jervaeus A., Lampic C., Eriksson L.E., Widmark C., Armuand G.M., Malmros J., Marshall Heyman M., Wettergren L. (2014). ‘Will I be able to have a baby?’ Results from online focus group discussions with childhood cancer survivors in Sweden. Hum. Reprod..

[bb0105] Nilsson S., Segerstad Y.H.A., Olsson M. (2022). Visualizing the invisible - the needs and wishes of childhood cancer survivors for digitally mediated emotional peer support. Curr. Oncol..

[bb0110] Olsson M., Enskär K., Steineck G., Wilderäng U., Jarfelt M. (2018). Self-perceived physical attractiveness in relation to scars among adolescent and young adult cancer survivors: A population-based study. J. Adolesc. Young Adult Oncol..

[bb0115] Pingree S., Hawkins R., Baker T., Dubenske L., Roberts L.J., Gustafson D.H. (2010). The value of theory for enhancing and understanding e-health interventions. Am. J. Prev. Med..

[bb0120] Rose S.R., Horne V.E., Howell J., Lawson S.A., Rutter M.M., Trotman G.E., Corathers S.D. (2016). Late endocrine effects of childhood cancer. Nat. Rev. Endocrinol..

[bb0125] Ryan R.M., Deci E.L. (2000). Self-determination theory and the facilitation of intrinsic motivation, social development, and well-being. Am. Psychol..

[bb0130] Schover L.R., Strollo S., Stein K., Fallon E., Smith T. (2020). Effectiveness trial of an online self-help intervention for sexual problems after cancer. J. Sex Marital Ther..

[bb0135] van Dijk M., van den Berg M.H., Overbeek A., Lambalk C.B., van den Heuvel-Eibrink M.M., Tissing W.J., Kremer L.C., van der Pal H.J., Loonen J.J., Versluys B., Bresters D., Kaspers G.J.L., van Leeuwen F.E., van Dulmen-den Broeder E., <collab>Group D.L.-V.S.</collab> (2018). Reproductive intentions and use of reproductive health care among female survivors of childhood cancer. Hum Reprod.

[bb0140] Vanstone R.N., Fergus K., Ladhani N.N.N., Warner E. (2021). Reproductive concerns and fear of cancer recurrence: a qualitative study of women's experiences of the perinatal period after cancer. BMC Pregnancy Childbirth.

[bb0145] Anon (2011). What should the age range be for AYA oncology?. J. Adolesc. Young Adult Oncol..

[bb0150] Wiljer D., Urowitz S., Barbera L., Chivers M.L., Quartey N.K., Ferguson S.E., TO M., Classen C.C. (2011). A qualitative study of an internet-based support group for women with sexual distress due to gynecologic cancer. J. Cancer Educ..

[bb0155] Winterling J., Wiklander M., Obol C.M., Lampic C., Eriksson L.E., Pelters B., Wettergren L. (2016). Development of a self-help web-based intervention targeting young cancer patients with sexual problems and fertility distress in collaboration with patient research partners. JMIR Res. Protoc..

[bb0160] Wootten A.C., Meyer D., Abbott J.M., Chisholm K., Austin D.W., Klein B., McCabe M., Murphy D.G., Costello A.J. (2017). An online psychological intervention can improve the sexual satisfaction of men following treatment for localized prostate cancer: outcomes of a randomised controlled trial evaluating My Road Ahead. Psychooncology.

[bb0165] Zebrack B.J., Casillas J., Nohr L., Adams H., Zeltzer L.K. (2004). Fertility issues for young adult survivors of childhood cancer. Psychooncology.

